# Differences between musicians and athletes in personal characteristics: mental toughness, imagery and personality

**DOI:** 10.3389/fpsyg.2025.1506123

**Published:** 2025-02-19

**Authors:** Dagmara Budnik-Przybylska, Izabela Huzarska-Rynasiewicz, Paweł Jurek, Jacek Przybylski

**Affiliations:** ^1^Division of Sport Psychology, Institute of Psychology, Faculty of Social Sciences, University of Gdańsk, Gdańsk, Poland; ^2^Faculty of Physical Culture Sciences, Medical College of Rzeszow University, Rzeszów University, Rzeszów, Poland; ^3^Division of Social Psychology, Institute of Psychology, Faculty of Social Sciences, University of Gdańsk, Gdańsk, Poland

**Keywords:** mental toughness, imagery, personality, musicians, athletes

## Abstract

**Introduction:**

It appears that music and sports share many common elements. However, it turns out that mental toughness and imagery are present in both musicians and athletes, but they are understood and conceptualized differently within these domains. The aim of the study was to explore the relationship between mental toughness, personality, and imagery in two unique groups.

**Methods:**

The sample consisted of a group of athletes (*N* = 164, 82 females and 82 males) and a group of musicians (*N* = 97, 61 females and 36 males), aged between 13 and 46 (*M* = 23.65; Median = 21), with varying levels of experience. The participants filled in the Imagination in Sports Questionnaire, the Short Scale of Mental Toughness in Sport Questionnaire-19, and the Big Five Inventory—Short, with indicators adjusted to both research groups. The results proved significant differences between the two groups.

**Results:**

Our findings indicated that musicians tend to exhibit lower levels of mental toughness compared to athletes. Additionally, musicians demonstrated reduced conscientiousness but higher levels of neuroticism and openness to experiences, as opposed to athletes. In the group of musicians, mental toughness was associated with lower extraversion, neuroticism, higher levels of agreeableness, and lower physiological feelings in imagery. Mentally tough athletes were characterized by lower neuroticism and higher situational and general imagery. Personality and imagery explained 21% of the variance in mental toughness among athletes and 37% among musicians. Neuroticism was the main predictor in both groups. Imagery, however, predicted mental toughness only in the athletes’ group.

**Discussion:**

The comparison of the unique groups brings a fresh perspective on theoretical and practical work in terms of individual differences, confirming the need of creating mental toughness-building interventions in mental preparation for performance in both music and sports.

## 1 Introduction

Music and sports share similarities, such as the time it takes to develop expertise (Hatfield, 2024), high performance environment (Bateman, 2014), and pressure during competition (John et al., 2019; Kaleńska-Rodzaj, 2023; Tan et al., 2024; Weinberg and Gould, 2023; Williamon and Antonini Philippe, 2020). Both sports and music support the development of cognitive abilities such as mental rotations (Pietsch and Jansen, 2012). The performances involve psychological stress which can cause a decrease in the quality of presenting trained skills (Baumeister, 1984). Furthermore, arousal increases before performance in both sports and music settings (Bartel and Thompson, 2021; Weinberg and Gould, 2023). Sports and music require implementing self-regulation strategies (Kegelaers et al., 2022). Excellence in performance has a similar meaning in sports and music (Hatfield, 2024). There are also noticeable differences between music and sport, for example in the definition of excellence. In music, the criteria tend to be more subjective, while in sports, success is typically measured by scores and timing, with an emphasis on quantity (Habe et al., 2019).

### 1.1 The concept of mental toughness in sports and music

Given the challenges that come with performing, both musicians and athletes should be resilient to stressors (Hatfield, 2024). Mental toughness can exist naturally as an inherited trait or be acquired through experience. Gucciardi et al. (2009, p. 67) claim that “Mental toughness is a collection of experientially developed and inherent sports specific and sport-general values, attitudes, emotions, and cognitions that influence how an individual approaches, responds to and appraises both negatively and positively construed to pressures, challenges, and adversities to consistently achieve his or her goals.”

Mental toughness has been studied as an important individual difference factor. It allows individuals to cope effectively with challenges and to persist under pressure (Clough et al., 2002; Gucciardi et al., 2015; Gucciardi, 2017). Mental toughness has the greatest impact in sports but is also acknowledged across a variety of other fields. This term releases positive psychological resources, which are important to achieve the best performance (Clough et al., 2002; Gucciardi et al., 2015) and effective coping with stressors (St Clair-Thompson et al., 2015; Aditya et al., 2024).

Mental toughness relates to positive experiences in childhood. Those who had experienced fewer negative events as a child proved to have higher mental toughness and well-being (Shaw et al., 2022). Research has shown that there is a genetic predisposition to higher levels of mental toughness (Horsburgh et al., 2009). Furthermore, the previous studies suggests that it can also be linked to personality traits (Gucciardi et al., 2009). Mental toughness correlates negatively with neuroticism and positively with the rest of the Big Five subscales (Horsburgh et al., 2009). Mental toughness is related to extraversion in individuals who train mountain climbing, which is a high-risk discipline (Egan and Stelmack, 2003). Personality traits can contribute to competition under pressure (Allen et al., 2013), which is consistent with mental toughness. Some disciplines force the development of a high level of mental toughness and specific configuration of personality traits. For instance, athletes who completed ultra-endurance marathons exhibited higher levels of extraversion and openness to experiences compared to population norms, as well as greater mental toughness (Goddard et al., 2019). Previous research has demonstrated a relationship between risk-taking attitudes and mental toughness, with a positive correlation to physical risk. There is a correspondence between mental toughness and performance, those athletes who were mentally tougher reported to experience flow more frequently (Crust and Swann, 2013; Lange, 2024).

Mental toughness is a factor that is not only relevant for sports activities but also for other life activities, because it is based on experiences with the environment (Lin et al., 2017). It is strongly connected with athletes’ expertise and successful performance (Jones et al., 2007). Also, it can be developed in sport-specific scenarios which athletes have to face and struggle with (Gucciardi et al., 2008). Mental toughness develops naturally through participation in sports competitions. Athletes must embrace challenges and endure hardship in their disciplines (Tibbert and Andersen, 2014). Mental toughness can be developed in specific conditions arising from a discipline context (Coulter et al., 2018). Mental toughness in athletes seems to be a protective factor in maintaining mental health and it is positively associated with achieving goals (Gucciardi et al., 2017). Through performing music, it is possible to enhance mental toughness as well (Lewis and Oney, 2014). Several mental toughness models have been developed so far (e.g., Clough et al., 2002; Jones et al., 2002; Gucciardi et al., 2008, 2015). Firstly, Clough et al. (2002) drew from hardiness theory to develop a multidimensional model of mental toughness. Secondly, Gucciardi et al. (2015) drew from theories of stress to develop a unitary model of mental toughness. These models differ in several aspects, but they also share some features; for example, self-belief is at the core of most definitions (e.g., Clough et al., 2002; Bull et al., 2005; Thelwell et al., 2005; Gucciardi et al., 2008).

However, the construct of mental toughness is still unexplored in the music environment (Kosirnik et al., 2022), very often described in academic literature as resilience (Kegelaers et al., 2021). In music, the subject of resilience is raised in a more generic way than in sports. Holmes (2017) highlighted that a primary challenge for musicians is the pursuit of a successful and enduring professional career. Additionally, they often encounter various environmental issues and are exposed to pressure while developing their professional paths. Mental toughness in a field of performing art such as music usually is considered in the context of mental health (John et al., 2019). Based on research literature, scholars propose to use mental skills training or emotion regulation. Therefore, it is recommended for musicians to build their psychological mental skills in order to withstand their music-specific stressors (Allan, 2016; Kaleńska-Rodzaj, 2023). However, the Mahoney et al. (2014) study showed that adolescent musicians build their mental toughness in a similar way as athletes, which is helpful in a good performance. The study on music students reported that the higher level of resilience is connected to higher self-efficacy (Arbinaga, 2023). What is more, Osborne et al. (2014) declare that resilience is an important psychological trait which can be helpful when encountering stressors related to music performance. Kegelaers et al. (2021) showed a strong unbeneficial connection between resilience and anxiety in musicians. It is another confirmation of the need for resilience-building exercises for this professional group (Araújo et al., 2017; Wiggins, 2011).

### 1.2 Imagery

As it was proved in the previous research, imagery use can predict mental toughness in athletes (Mattie and Munroe-Chandler, 2012). Also, other studies stressed the aspect of the connection between imagery and mental toughness, mainly between the motivational sub-dimension of the imagery and mental toughness (Guerin et al., 2014; Mattie and Munroe-Chandler, 2012; Yalçın et al., 2022). Athletes may use imagery to cope with psychological difficulties connected to training and competitions, work with self-confidence to predict the outcome of their actions (Yalçın et al., 2022). Moreover, imagery techniques are used to build mental toughness (Clough et al., 2002; Ersin and Tutal, 2024). The imagery ability is necessary in different kinds of human activities. It is defined as “creation or re-creation of an experience generated from memorial information, involving quasi-sensorial, quasi-perceptual, and quasi-affective characteristics. This ability is under the volitional control of the imager, and which may occur in the absence of the real stimulus antecedents normally associated with the actual experience” (Morris et al., 2005, p. 19). It has been proven that people who practice sports regularly present a better imagery ability than novices or non-athletes (Cumming and Williams, 2013). Imagery ability also influences imagery usage. The research supports that the motivational nature of imagery ability – like goal, effect, and mastery (Cumming and Williams, 2012) – and a general tendency to use imagery in daily life are not only positive predictors of athletes’ sport confidence (Budnik-Przybylska et al., 2022) and response to anxiety, but also of challenge and threat stress appraisal tendencies (Cumming and Williams, 2012). Moreover, mental toughness and imagery are significant predictors of athletes’ somatic anxiety (Budnik-Przybylska et al., 2018).

Imagery ability in musicians is a multimodal process that facilitates the planning of actions to synchronize musical elements and movement (Keller, 2012). In the context of modalities, the most used senses by athletes are sight and kinesthesia (Budnik-Przybylska et al., 2014; Morris et al., 2005; Lotze, 2013); otherwise, musicians have a stronger auditory imagery (Clark et al., 2012; Talamini et al., 2023). Music performance is by definition multimodal (Küssner et al., 2024). Musicians’ imagery skills include both imagining sounds and individual body movements while playing (Clark et al., 2012). While imagining a musical performance, musicians first activate the auditory channel, and only later or less intensely the motor movements (Bastepe-Gray et al., 2020; Meng and Luck, 2024). Interestingly, the auditory modality can influence the development of movement skills. It is suggested to start an imagery training from dominant sense (Jenny et al., 2015). Music students frequently use imagery to mentally rehearse performance (Bailes, 2007). Imagery in music mainly involves the ability to imagine sounds, even without hearing them. With this method, musicians can envision all elements necessary for a successful performance, not only the sounds. This includes the physical movements needed to produce the sound, the sight of the score or instrument, and the emotions they wish to convey (Clark et al., 2012).

It was found that musicians reported using imagery to cope with distractions, recover from errors, maintain mental toughness, build self-confidence, overcome fatigue – both mental and physical (Gregg and Clark, 2007; Gregg et al., 2008) – and also achieve a high standard of proficiency involving complex cognitive elements (Bernardi et al., 2013) and a high degree of motor control (Watson, 2006). What is more, openness and neuroticism can help predict the imagery ability of musicians (Beaty et al., 2013). As a personal trait, anxiety is positively correlated with involuntary imagery of disturbed images, as well as a helpful constructive imagery of musicians (Ulor et al., 2022). There is an existing link between openness and general imagery ability which can be measured (Sassenberg et al., 2023; Budnik-Przybylska et al., 2019) and connected to divergent thinking and creativity (DeYoung et al., 2012).

### 1.3 Personality

The performance of various motor activities such as postural balance can be linked to specific personality traits like openness and emotional reactivity (Wojciechowska-Maszkowska et al., 2020). Moreover, higher-level athletes are more conscientious, compassionate, and emotionally stable than lower-level athletes. Additionally, athletes who are extroverted, emotionally stable and open to new experiences, can be also characterized by a greater use of problem-focused coping strategies (Allen et al., 2011). Musicians more often have a higher level of openness compared to non-musicians, but lower conscientiousness (Gjermunds et al., 2020). Neuroticism has been linked to certain aspects of performance deterioration, as studied in the sample of 258 pianists (Furuya et al., 2021).

Based on the aforementioned literature, we decided to delve deeper into the relationship between mental toughness, personality, and imagery in two unique groups. Previous studies revealed a gap in examining the levels of imagery, mental toughness, and personality in musicians within the performance context. We are aware that this knowledge is more widely analyzed in the groups of athletes and provides a better understanding of athletes’ functioning. Taking this into account, we recognized the need to apply this knowledge to the field of music.

After an in-depth analysis, several hypotheses were formulated.

*H1*: We predicted that there were differences in mental toughness, imagery, and personality between athletes and musicians. Athletes had a higher level of mental toughness, imagery ability and, as for personality, had a higher extraversion and conscientiousness but lower neuroticism.

*H2*: The associations between mental toughness and both personality and imagery varied across the analyzed groups. We expected the mental toughness to be more linked to lower levels of neuroticism, as well as all subscales of imagery in the group of athletes, in comparison to musicians.

*H3*: Imagery and personality were identified as predictors of mental toughness in both analyzed groups. All subscales of imagery and mainly neuroticism would be predictors of mental toughness.

## 2 Materials and methods

### 2.1 Participants and procedure

In the study, participants were invited to take part as volunteers. There was a group of athletes (*N* = 164, 82 females and 82 males) and musicians (*N* = 97, 61 females and 36 males), aged between 13 and 46 (*M* = 23.65, SD = 6.30, Me = 21; for athletes: *M* = 25.84, SD = 6.91, Me = 24; for musicians: *M* = 19.95, SD = 2.19, Me = 19), with different levels of experience between 0,5 to 32 years (*M* = 10.67; SD = 5,51; Me = 10; for athletes: *M* = 10.10, SD = 6.63, Me = 9; for musicians: *M* = 11.64, SD = 2.55, Me = 12). The majority of the participants were emerging adults (emerging adulthood described as typically lasting between the age of 18 and 29) (Willoughby et al., 2021). Among the athletes were representatives of various disciplines, for instance, handball, tennis, martial arts, and swimming. Musicians declared to play a variety of instruments like the violin, flute, harp, piano, and guitar. Participants represented the university and national levels of experience.

We have made every effort to adjust the measurements for both groups. The priority was to understand and adapt the items to the groups’ context. Taking all into account, the nomenclature was adapted for each group without changing the meaning of all sentences.

The research was conducted by the first and the fourth of the listed authors. Athletes were invited to the study during lectures at university and during sports trainings, musicians filled in the questionnaires during music classes at music school. The questionnaires were presented on paper in the same order for each participant. They filled out questionnaires with no time limits.

Written consents were obtained from the musicians and the athletes to participate in the study and personal data protection was properly secured. In the case of minors, the parental consents were obtained from their legal guardians. The research followed the ethical principles regarding human experiments as defined in the Declaration of Helsinki, and the study was approved by the local Institutional Review Board (*protocol number:* 11/2015).

### 2.2 Research tools

#### 2.2.1 Imagery

To measure imagery the Imagination in Sport Questionnaire was applied (Budnik-Przybylska, 2014). It is a multi-dimensional tool that consists of 51 statements in seven aspects: physiological feelings (6 questions) (noticeable changes in the way the body works), modalities (7 questions) (using different sense modalities except sight), ease/control (10 questions) (ease of generating images and control over imagined situations), perspective (8 questions) (ability to adopt different perspectives), affirmations (8 questions) (positive attitude during a competition), visual (6 questions) (using sight in imagery) and general scale (6 questions) (general tendency to use imagination). For 60 s, the respondent imagines a hypothetical start in a competition after which they evaluate the statements in the questionnaire concerning different imagery aspects on a scale from 1 to 5, with one meaning “not at all” and five, “absolutely yes.” All the subscales (except for the “general” subscale) were related to the imagined situation, i.e., situational imagery. The “general” subscale consisted of six questions and was developed separately to assess the general tendency to use imagery. The study items for musicians were updated to hypothetical musical performances instead of participating in sports competitions. The exact instructions for the music group were:

“Imagine yourself before an important performance. Spend about 60 s on this task. If you want to you can close your eyes. Try to make the imagination as real as possible, have as many details as possible, and pay attention to all the elements. Imagine what you see, what you hear, what you feel, what you are doing, what others are doing, and what is happening around you. Feel the emotions and sensations that this situation exerts on you. Then rate each aspect of your imagination on a scale of 1 to 5 by marking next to each statement with the corresponding number, where 1 means ‘not’ at all and 5 means ‘completely yes’.”

We calculated Cronbach’s alpha for both groups. The results for the musicians are as follows: physiological feelings: 0.84, modalities: 0.66, ease/control: 0.81, perspectives: 0.79, affirmations: 0.87, visual: 0.79, general: 0.66, and for athletes: physiological feelings: 0.85, modalities: 0.70, ease/control: 0.86, perspectives: 0.82, affirmations: 0.87, visual: 0.65, general: 0.79.

For a better understanding of the usage of this questionnaire, we provide samples of items assigned specifically to athletes and musicians. For the subscale, the perspective for athletes was: *Can you imagine this event from the perspective of the coach?* While for musicians: *Can you imagine the event from the perspective of the person who prepared you for the performance?* As for ease/control: *How easily can you develop the tactics in your imagination?* Whereas for musicians: *How easily can you develop the course of the performance in your imagination?* For the subscale affirmations, the original statement was: *You tune positively to a successful start.* The musicians read: *You tune positively to a successful performance*.

Unchanged remained i.a. physiological feelings: *How clearly did you feel the emotions that you experienced?* For modalities: *How clearly did you hear the sounds occurring in this situation? For visual: Were the colors occurring in this situation clear?* For general: *Do you imagine the events ahead for you?*

#### 2.2.2 Mental toughness

The Short Scale of Mental Toughness in Sport Questionnaire-19 (SMTSQ-19), is a newer and tailored version of the original 42-item questionnaire, which previously had three subscales. Shortening the 42-factor version of the MTSQ (Przybylski, 2018) and psychometrically testing the shortened 19-factor version of the SMTSQ was a basic requirement for improving the questionnaire’s performance. The new version, characterized by the selected 19 factors, does not have scales as in the original version. The total sum of the questionnaire scores calculates the final score. The higher the intensity of the trait, the lower the body’s resistance to stressors and lower mental toughness. The instruction was constructed in the following way:

“Decide which of the following factors appear in your life and to what extent they stress you, irritate you, bother you, or annoy you. Determine the severity of each of these factors on a scale of 0–10. An answer of 0 means that the given factor is completely neutral or absent to you. An answer of 1 means that these factors stress you or annoy you very little. An answer of 10 means that the factor stresses you or annoys you at a very high level.”

Since this new tool was not published before, the author provides psychometric data for this measure. One factor solution provides a sufficient fit of the measurement to data [*χ*^2^(819) = 5508.504; RMSEA = 0.106; CI95 = (0.104;0.109); SRMR = 0.084; TLI = 0.860; CFI = 0.867]. For musicians all items concerning sports were changed to musical performance. Cronbach’s alpha for the group of musicians was 0.91, while for athletes it was 0.93.

For a better understanding of the usage of this questionnaire, we provide samples of items assigned specifically to athletes and musicians. For athletes there was *an unclear evaluation style of your coach,* while for musicians, *an unclear evaluation style of your teacher*; then, for athletes, *fear of failure* and for musicians, *fear of negative appraisal*; next, for athletes, *poor training’s effects*, while for musicians, *poor practice’s effects*.

#### 2.2.3 Personality

To study personality, the Polish version (Strus et al., 2017) of the Big Five Inventory—Short (BFI-S, Gerlitz and Schupp, 2005) was used. It is a 15-item tool with a seven-point scale of answers, where 1 means “definitely not” and 7 “definitely yes.” This tool is used to measure personality in terms of a five-factor personality theory. Specific subscales measure the following traits: extraversion, openness to experience, agreeableness, neuroticism, and conscientiousness. Due to its short form, this scale is increasingly being used in exploratory research measuring many variables (Hahn et al., 2012; Lang et al., 2011). Cronbach’s alpha for the group of musicians was the following: neuroticism: 0.69, extraversion: 0.57, openness to experience: 0.77, agreeableness: 0.66, conscientiousness: 0.76 while for athletes: neuroticism: 0.59, extraversion: 0.43, openness to experience: 0.58, agreeableness: 0.53, conscientiousness: 0.69.

### 2.3 Statistical analysis

We calculated the average and standard deviations of the variables tested. For direct group comparisons, a *t*-test was employed. Pearson correlation was used to illustrate the associations between factors. Linear regression analyses were conducted to identify the predictors of mental toughness. The significance level was set at *α* = 0.05. The IBM SPSS Statistics version 27 was used to perform the calculations. The study design and analysis were not preregistered.

## 3 Results

First, we examined the differences in mental toughness, imagery, and personality between the athletes and musicians. The results are presented in Table 1.

**Table 1:** Differences between musicians and athletes in analyzed scales.

	Musicians		Athletes		*t*	*df*	*p*
	*M*	*SD*	*M*	*SD*			
Mental toughness (the higher the level the lower mental toughness)	100.16	39.72	89.74	41.95	1.98	259	0.05
Openness to experience	5.51	1.11	5.23	1.13	1.92	259	0.06
Conscientiousness	4.53	1.32	5.48	1.14	−6.12	259	0.00
Extraversion	4.02	1.31	4.06	1.17	−0.22	259	0.82
Agreeableness	5.03	1.22	4.88	1.17	1.01	259	0.31
Neuroticism	4.57	1.44	4.14	1.33	2.46	259	0.01
Physiological feelings	20.41	5.68	22.94	5.13	−3.69	259	0.00
Modalities	18.38	5.16	19.38	5.31	−1.48	259	0.14
Ease/control	36.93	6.59	39.70	6.46	−3.33	259	0.00
Perspective	26.56	6.30	27.68	6.38	−1.39	259	0.17
Affirmations	27.81	6.83	33.45	5.31	−7.43	259	0.00
Visual	21.91	4.84	23.62	3.78	−3.19	259	0.00
General	24.34	3.40	25.13	3.57	−1.75	259	0.08

We observed significant differences between musicians and athletes on the following scales: mental toughness, conscientiousness, neuroticism, physiological feelings, ease/control, affirmations, and visual. Marginal significance we observed in openness to experience and general subscales (openness *p* = 0.06, general *p* = 0.08). Musicians achieved higher scores on the SMTSQ 19, indicating that they exhibit a lower level of mental toughness when compared to athletes. Regarding personality, musicians scored higher than athletes in openness to experience and neuroticism but lower in conscientiousness. In ISQ musicians obtained lower scores in all significant differences, specifically in physiological feelings, ease/control, affirmations, and visual.

In the next step, we addressed the second hypothesis regarding the associations between mental toughness, personality, and imagery in the analyzed groups. First, for each dimension of personality and imagery, we fitted an interaction model (predictor x professional group) for the entire sample to identify statistically significant moderation effects. Next, we conducted separate regression analyses for the two professional groups. In all models, the length of experience was included as a control variable. The results are presented in Table 2.

**Table 2:** Relationship between specific dimensions of personality, imagery, and mental toughness, controlling for length of experience in musicians and athletes: results of regression analysis with interaction (total sample) and separate analyses for two professional groups.

Predictor	Results of the Interaction analysis (predictor × Professional group)		Results of the regression analysis conducted separately in professional groups			
			Musicians		Athletes	
	*B* (*SE*)	Beta	*B* (*SE*)	Beta	*B* (*SE*)	Beta
Openness to experience	8.75 (4.71)	0.58	3.46 (3.63)	0.09	0.64 (0.49)	−0.15
Conscientiousness	1.30 (4.25)	0.07	−1.50 (3.07)	−0.05	−2.84 (2.87)	−0.08
Extraversion	9.18 (4.19)	0.47*	2.14 (1.55)	0.23*	−2.25 (2.18)	−0.06
Agreeableness	−9.07 (4.38)	−0.56*	−6.74 (3.26)	−0.21*	2.12 (2.81)	0.06
Neuroticism	1.50 (3.50)	0.08	12.37 (2.52)	0.45**	11.01 (2.33)	0.35**
Physiological feelings	1.52 (0.96)	0.39	1.83 (0.69)	0.26**	0.32 (0.64)	0.04
Modalities	0.84 (1.01)	0.19	0.96 (0.78)	0.13	0.07 (0.62)	0.01
Ease/control	1.53 (0.80)	0.68	0.21 (0.61)	0.04	−1.36 (0.50)	−0.21**
Perspective	0.28 (0.81)	0.09	−0.53 (0.65)	−0.08	−0.90 (0.51)	−0.14
Affirmations	2.11 (0.83)	0.72*	−0.65 (0.59)	−0.11	−2.74 (0.58)	−0.35**
Visual	1.81 (1.20)	0.48	−0.09 (0.84)	−0.01	−19.96 (0.86)	−0.18*
General	2.80 (1.51)	0.81	0.18 (1.19)	0.02	−2.73 (0.90)	−0.23**

*p < 0.05, **p < 0.01.

As shown, significant interaction effects were observed for extraversion, agreeableness, and affirmations. In the musicians’ group, extraversion was a significant positive predictor, while agreeableness was a significant negative predictor of mental toughness. In contrast, these personality traits were not statistically significantly associated with mental toughness in the athletes’ group. However, in the athletes’ group, affirmations emerged as a significant negative predictor, which was not observed among musicians.

Additionally, in the musicians’ group, significant correlations between mental toughness, personality and imagery were found, that is, positive correlations with neuroticism and physiological feelings. In the athletes’ group, we observed significant correlations between mental toughness and personality and imagery, specifically, a positive correlation with neuroticism and negative correlations with all subscales of imagery (ease/control, perspective, visual, and general). However, no significant interaction effects were noted for these variables.

As the last step, we tested the third hypothesis concerning imagery and personality as predictors of mental toughness in both analyzed groups. We used the backward stepwise method and incorporated gender and experience into the models. Table 3 presents the results of the baseline and final models for each professional group separately.

**Table 3:** Results of the full and final models for mental toughness prediction: separate analyses for each professional group.

Predictor	Musicians				Athletes			
	Baseline model		Finale model		Baseline model		Finale model	
	*B* (*SE*)	Beta	*B* (*SE*)	Beta	*B* (*SE*)	Beta	*B* (*SE*)	Beta
Intercept	65.46 (50.28)	–	49.86 (21.17)	–	97.77 (37.38)	–	85.94 (28.60)	–
Gender (male)	−28.02 (7.55)	−0.34**	−27.98 (7.00)	−0.34**	−5.99 (6.66)	−0.07		
Age	0.56 (1.82)	0.03			0.01 (0.49)	0.01		
Experience	1.00 (1.46)	0.06			0.80 (0.49)	0.13	0.78 (0.45)	0.12
Openness to experience	0.09 (3.49)	0.01			−0.96 (3.30)	−0.03		
Conscientiousness	−0.07 (2.78)	−0.01			−0.60 (3.00)	−0.02		
Extraversion	7.99 (2.95)	0.26**	7.28 (2.58)	0.24**	−2.55 (2.90)	−0.07		
Agreeableness	−2.54 (2.97)	−0.08			5.16 (2.70)	0.14	5.16 (2.69)	0.14
Neuroticism	7.29 (2.84)	0.26*	9.40 (2.36)	0.34**	8.06 (2.66)	0.26**	7.88 (2.46)	0.25**
Physiological feelings	0.56 (0.83)	0.08			0.87 (0.76)	0.11	1.22 (0.64)	0.15
Modalities	0.99 (0.87)	0.13	1.10 (0.65)	0.14	0.68 (0.67)	0.09		
Ease/control	0.54 (0.77)	0.09			0.05 (0.87)	0.01		
Perspective	−0.57 (0.71)	−0.09			−0.03 (0.68)	−0.01		
Affirmations	−1.02 (0.61)	−0.18	−1.14 (0.51)	−0.20*	−2.49 (0.90)	−0.32**	−2.66 (0.66)	−0.34**
Visual	−0.41 (0.87)	−0.05			−0.69 (1.09)	−0.06		
General	−1.13 (1.33)	−0.10			0.50 (1.29)	0.04		
Adjusted *R*^2^	0.33		0.37		0.17		0.21	

*p < 0.05, **p < 0.01.

As shown, the regression analysis revealed that imagery and personality accounted for 37% of the variance in mental toughness [Adjusted *R*-squared = 0.37, *F*(5,91) = 12.36, *p* < 0.001] in the musicians’ group, and 21% of the variance in mental toughness [Adjusted *R*-squared = 0.21, *F*(5,157) = 9.35, *p* < 0.001] in the athletes’ group. Specifically, the contribution of each subscale of imagery and personality to explaining mental toughness as the dependent variable in the musicians’ group showed that the significant predictors were extraversion, neuroticism, and affirmations. Additionally, in this group, males scored lower on the mental toughness scale (indicating higher mental toughness). For the athletes’ group, the significant predictors of mental toughness were neuroticism, agreeableness, and affirmations.

## 4 Discussion

The concept of mental toughness in athletes is well known (Gucciardi et al., 2015), but there are not many studies about mental toughness in musicians. The concept of mental toughness may encompass different factors for athletes and musicians, as these individuals interact with their environments in distinct ways. The study presents new insights into individual differences in the combined use of mental skills.

As the first step, we hypothesized that there would be differences in mental toughness, imagery, and personality between athletes and musicians. We assumed that athletes have a higher level of mental toughness, imagery ability and as personality is concerned, they will have higher extraversion and conscientiousness but lower neuroticism. Our findings confirmed the expected differences in mental toughness and conscientiousness; however, extraversion did not differ significantly between groups. The lower mental toughness observed in musicians may stem from differences in training routines and self-control strategies commonly developed in athletic training (Englert, 2016). It also might be connected to musicians creating less extensive social networks (John et al., 2019) and their dissatisfaction with music environment conditions (Pecen et al., 2018). The athletes can be also more goal-oriented; however, musicians might be more focused on feelings (Habe et al., 2019). Our finding confirmed the need of resilience-building interventions to help musicians perform better (Kegelaers et al., 2021; Araújo et al., 2017).

Moreover, compared to athletes, musicians exhibited lower levels of conscientiousness higher levels of neuroticism and openness to experience (with marginal significance), aligning with previous research (Gjermunds et al., 2020). Otherwise, in extraversion, there were no significant differences between groups. Musicians presented a higher tendency toward anxiety, negative feelings, and self-doubt, with a worse response to stressors and a higher emotional instability than athletes. Differences in neuroticism could be explained in the following way: sports athletes should turn off emotions in their performance or direct their attention away from emotional stimuli (Janelle et al., 2020). Adversely, musicians express emotions of musical pieces on stage, therefore they were more emotionally reactive. So, it also might relate to musical habits such as music sensibility or emotion regulation (Miranda, 2020). More frequent factors of anxiety might be also connected to a highly competitive music environment (Biasutti and Concina, 2014) and subjective music performance evaluation (Habe et al., 2019).

As was reported in the Pecen et al. (2018) study, musicians use psychological coping strategies out of purpose, intuitively, rather than as a way to solve challenges. This may be the reason why they focus more on emotions. Therefore, sports psychologists should work with musicians to enhance their psychological skills and help them achieve an optimal mental state for performance (Connolly and Williamon, 2004). Mental training is a process in which athletes (more often also musicians), usually with the help of consultants or coaches, systematically use strategies and techniques to build mental skills and wellbeing to enhance their performance, development, and experiences (Vealey, 2024).

While musicians demonstrated lower levels of conscientiousness, their higher openness to experience suggests a greater capacity for creative adaptation and innovation within their field. Conversely, athletes preferred a more structured routine, often associated with a training plan (Costa and McCrae, 1992). Openness to experience is linked to the flexibility of imagined situations, ease of assimilation, and having a vivid and creative imagination. This also tends to go beyond the framework of a chosen field, ease of processing and assimilation of new information. Furthermore, it links with a higher demand for new stimuli and unusual experiences. Surprisingly, we did not discover any differences in an extraversion level between the groups, which may be due to the excessive heterogeneity of the group of musicians as representatives of different instruments (Gjermunds et al., 2020).

While athletes achieved higher scores in most scales of imagery apart from modality where musicians obtained similar score. The modality subscale is linked to the use of all modalities apart from the visual one which is a separate subscale. Previous studies focused on music imagery in musicians such as hearing and playing, recreating music in their heads (Cotter et al., 2019). Some studies focus on the use of imagery rather than the ability itself (Clark et al., 2012). Sound imagery is crucial for memorization in music, assisting in the retention of its many elements (Gregg et al., 2008). Because of the complexity of the music-making process, imagining and analyzing a piece of music with all senses before instrument practice is also beneficial. This approach allows one to focus on the music itself (Holmes, 2005).

Athletes were more prone to use imagery in the sense of being more aware of their bodies in the context of physiological feelings. They had more images and controlled them more easily, appeared more self-confident in imagined situations, and used visual imagery more frequently (Morris et al., 2005). On the other hand, as we mentioned in the introduction, musicians have greater ease of imaging when it comes to just auditory channel (Talamini et al., 2023).

As the second assumption, we stated that there would be different associations between mental toughness, personality, and imagery in analyzed groups. We expected that mental toughness would be more linked to lower levels of neuroticism as well as all subscales of imagery in the group of athletes compared to musicians. This hypothesis was also confirmed. We found that, among musicians, mental toughness was associated with lower extraversion and neuroticism, higher agreeableness, and a lower level of physiological feelings in imagery. Moreover, a higher level of neuroticism was linked to performance deterioration in pianists (Furuya et al., 2021). A higher level of agreeableness is linked to better conflict resolution skills. This may be more necessary in the music community as an aspect of mental toughness. In imagery physiological feelings help to be more aware of body sensations. However too high a concentration of the body may lead to deterioration of automaticity and a stronger focus on emotions than on the task (Budnik-Przybylska and Kuchta, 2020). It is also shown that using physiological sensations during imagery such as changes in breath rate, emotional sensations at the start, and heightened awareness of body movement can contribute to increased performance anxiety. These sensations may heighten an individual’s awareness of their anxiety (Budnik-Przybylska et al., 2018). Similar findings were confirmed in the Monsma and Overby study (2004). It is also confirmed that a higher emotionality as the trait could disrupt imagery usage (Budnik-Przybylska et al., 2022).

The third hypothesis was that imagery and personality were the predictors of mental toughness in both analyzed groups. We assumed that all subscales of imagery and mainly neuroticism would be predictors of mental toughness. It was confirmed both in the athletes’ and musicians’ groups in the context of imagery. For the group of musicians, it turned out that extraversion, neuroticism and affirmations were the significant predictors. Similarly to the musicians’ group, for the athletes, the significant predictors of mental toughness were neuroticism and affirmations, but also agreeableness. Sports athletes use mental imagery more often to build mental toughness (Mattie and Munroe-Chandler, 2012). Our results were also in line with the study of Clough et al. (2002), stating that mental toughness correlates negatively with neuroticism. As personality factors, neuroticism was a significant and positive predictor in both groups, and extraversion was a positive predictor in musicians. The ability to imagine having a positive attitude to various situations seems to be a protective factor in coping with challenges (Cumming and Williams, 2012).

### 4.1 Limitations

Our study was not free from limitations. The genders in the musicians’ group should be more evenly balanced. However, in our study participated mostly representatives of the violin, flute, harp, and singers, all of which are more popular choices among females (Sergeant and Himonides, 2019). In the future studies, it would be worthwhile to control age and gender aspects. It is also advisable to focus on comparing musicians to athletes in closed-skill sports and those with a connection to music. It would also be valuable to compare solo performers and individual sports athletes with members of orchestras and team-sport athletes. This comparison could consider the different types of stress they may experience. The sports and music performances are similar in their forms because all moves are memorized and planned. In our research, we demonstrated that the predictors were consistent for both athletes and musicians. This shows that perhaps the group of musicians was too small, so the effect was not significant.

We are also aware that questionnaires on imagery and mental toughness used in this study were originally designed for sports. However, we tailored the items to be relevant for musicians and calculated the necessary statistics to assess the reliability of these measures in both groups. We made every effort to ensure the questionnaire was equally applicable to both research groups, considering the context familiar to each.

### 4.2 Strengths of the study

It is noteworthy to include performers like musicians in studies about mental toughness because this is still an unexplored area (Kosirnik et al., 2022). The study established a connection between specific sports, music performance, and individual differences. We demonstrated that, despite facing similar challenges, individual differences are significant across various aspects of life. It would appear that musicians and athletes face similar mental challenges, whereas there are differences in the use of techniques and differences in personality. We found that mental toughness may be associated with personality and imagery, but its development could be influenced by one’s profession. With some personality traits, a person fits the discipline and faces challenges in different ways of handling. We treated this study as an exploration of those analyzed concepts in two fields. We also looked for the linkage between two kinds of performance and direction for future studies.

### 4.3 Practical implications

The comparison of unique groups, a valuable insight into the topic mentioned, brings a fresh perspective on the theoretical and practical work. These valuable pieces of information can help practitioners adapt their working measures, taking the highlighted factors into account. We assume that musicians use imagery to work in technical aspects of music playing, not to enhance mental toughness. Nevertheless, they could still develop it for different purposes of music performance. The presented approach proves that this is a huge area to work with musicians on developing imagery to build mental toughness. It is also possible that athletes were more aware of the effectiveness of mental training techniques. Athletes showed how to strengthen their mental toughness, and this knowledge could be transferred to the world of music. Musicians’ emotionality seemed to be a good guideline to use in everyday life.

## Data Availability

The raw data supporting the conclusions of this article will be made available by the authors, without undue reservation.
